# Solar Energy Harvesting using Candle‐Soot‐Coated Thermoelectric Materials

**DOI:** 10.1002/gch2.201900080

**Published:** 2020-05-19

**Authors:** Deepshikha Yadav, Puneet Azad, Rahul Vaish

**Affiliations:** ^1^ Department of Electronics & Communication Engineering Maharaja Surajmal Institute of Technology New Delhi 110058 India; ^2^ University School of Information, Communication & Technology Guru Gobind Singh Indraprastha University New Delhi 110078 India; ^3^ School of Engineering Indian Institute of Technology Mandi Himachal Pradesh 175001 India

**Keywords:** candle soot, energy harvesting, solar power, thermoelectric power

## Abstract

This article reports the thermoelectric‐based solar energy harvesting. The effect of candle soot (CS) coating on solar energy harvesting potential of thermoelectric modules is studied. To compare the performance, uncoated/coated modules are exposed to solar radiations (through Fresnel lens) and the other side is kept at lower temperature using continuous water flow. Substantial enhancements in electrical outputs are observed due to CS coating on the upper surface of the thermoelectric module. The open‐circuit voltage and short‐circuit current across coated module improve more than six times in comparison to the uncoated module with maximum voltage and current reaching up to 1.5 V and 14 mA. Similarly, the generator can deliver a maximum power of 10 mW across a resistance of 50 Ω. Results indicate that the CS coating is an effective technique to improve the performance of thermoelectric materials for running sensors and other low‐power electronic devices.

## Graphical abstract

Significant improvements in performance of thermoelectric materials under solar radiations are observed due to candle soot (CS) coating over their surface. Electrical outputs such as open circuit voltage, current, and power improved multifold across coated modules. Results indicate that soot coating is an effective technique to improve the performance of thermoelectric materials for running sensors and other low‐power devices.

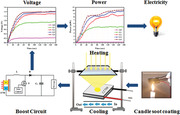

## 1 Introduction

Exploring new renewable energy sources (for low power electronics and portable devices) has been a key area of research to minimize the ever demanding need of batteries and ensuring continuous power supply. Energy harvesting techniques utilize untapped ambient energy from the environment and can convincingly address such issues. Many techniques such as pyroelectricity,^[^ ^1^, ^2^ ^]^ thermoelectricity,^[^ ^3^ ^]^ photovoltaic,^[^ ^4^ ^]^ piezoelectricity,^[^ ^5^ ^]^ triboelectricity,^[^ ^6^, ^7^, ^8^, ^9^, ^10^, ^11^ ^]^ etc., are extensively studied to capture and harness the energy from ambient sources. Hybrid integrated systems have also been developed for maximum utilization.^[^ ^12^ ^]^ Among all such techniques, thermoelectric energy generator (TEG) uses freely available heat energy from vehicles, pavements, sunrays, and power plants to work using Seeback effect.^[^ ^13^, ^14^ ^]^ The thermal energy can be converted to electrical energy due to temperature difference between two semiconductor‐based thermocouple. These light weight modules are noiseless, robust, and enjoy larger shelf‐life in absence of moving parts. The advent of small band gap semiconductors provided new efficient materials for further examination. Various researchers have attempted to increase the efficiency of the thermoelectric modules by reduction in thermal conductivity, increase in electrical conductivity, and improvement of Seeback coefficient.^[^ ^15^, ^16^ ^]^

Efficient utilization of solar energy for electricity generation has attracted greater attention in last few decades.^[^ ^17^ ^]^ Additionally, coatings of high‐performance heat absorption materials have elevated the contribution of solar energy toward energy harnessing. Extreme heat from sunlight easily creates the spatial temperature difference required for thermal heat conversion by thermoelectric modules. Collaboration of solar‐thermal effect has witnessed plenty of promising applications like water distillation, electricity generation, heating of water, etc.^[^ ^18^, ^19^, ^20^ ^]^ A brief discussion on ZnSb and BiSb alloys‐based solar thermoelectric generators was reported by Telkes.^[^ ^21^ ^]^ Xia et al.^[^ ^22^ ^]^ fabricated a bismuth telluride and carbon nanotubes‐based composites giving 1% efficiency in ambient sunlight. Cheruvu et al.^[^ ^23^ ^]^ analyzed a solar thermoelectric generator (STEG) giving maximum efficiency of 2.21% with output power of 0.91 W when placed in a vacuum enclosure. Kraemer et al.^[^ ^24^ ^]^ reported a flat‐panel‐type STEG with 4.6% peak, further improved by Goldsmid et al.^[^ ^25^ ^]^ using optically concentrated method. A 1.08 W matched load power is generated by Nia et al.^[^ ^26^ ^]^ with 51.33% efficiency using a polymethyl methacrylate (PMMA) Fresnel lens system for heat concentration.

Certain absorbing materials like carbon nanotubes, graphene oxide, etc., can increase the heat absorbance capacity to enhance the performance. Candle soot is a well‐known cost‐effective source of carbon nanoparticles. It gives stable results even at 500 °C^[^ ^27^ ^]^ and exhibits superhydrophobic property. Deposition of candle soot on surface shows favorable properties, like luminescence, in visible light spectrum.^[^ ^28^ ^]^ When concentrated sunlight falls on these highly absorbent materials, output power enhances greatly. Azad et al.^[^ ^29^ ^]^ reported a 50 times enhanced harvested energy using candle‐soot‐coated PZT pyroelectric device while diesel exhaust soot coating showed 17 times increase as reported by Azad et al.^[^ ^30^ ^]^ Ghasemi et al.^[^ ^31^ ^]^ proposed heat localization for steam generation using carbon‐based double‐layer solar‐thermal structure. An efficient TEG system not only demands an optimum temperature difference but also a matched load impedance condition. To draw maximum generated power, load resistance should match the internal impedance of TEG. Eakburanawat and Boonyaroonate^[^ ^32^ ^]^ connected three TEG modules with microcontroller‐controlled single ended primary inductance converter (SEPIC) DC–DC converter to charge a battery. 80% efficient TEG arrays with buck‐boost converter are reported by Nagayoshi and Kajikawa ^[^ ^33^ ^]^ for effective load conductance. Maximum power point‐tracked TEG with synchronous rectification was developed by Chen et al.^[^ ^34^ ^]^ transferring 47 W to a bulb. Kinsella et al.^[^ ^35^ ^]^ tested a single TEG to charge a Li‐ion battery using SEPIC converter. Azad^[^ ^36^ ^]^ reported 47.54 V boosted voltage across a 10 µF capacitor for a temperature difference of 56 °C. The above‐mentioned literature is the witness of multi‐dimensional attempts to improve energy harvesting performance of TEG. In order to study cost‐effective method to improve the performance of thermoelectric modules, the present study was planned. We exposed the candle flame to the upper surface of thermoelectric module (12 706) for efficient solar heat absorption. Further, temperature difference was maintained using solar radiations and continuous water flow at counter surfaces. The voltage was further boosted using a dc–dc boost converter to charge a 2.4 V VARTA NI‐MH rechargeable battery.

## 2 System Design and Description

**Figure 1:**
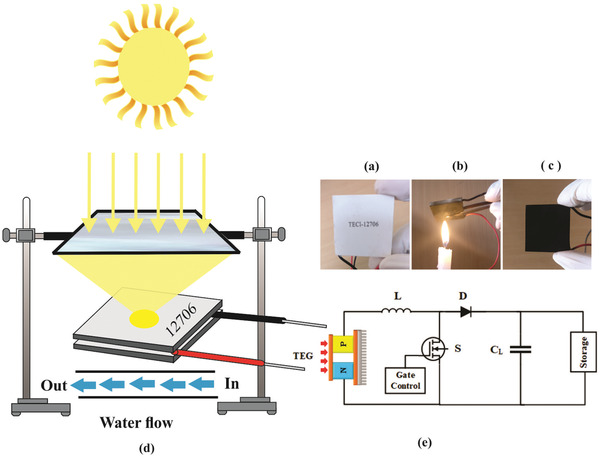
Schematic representation of the system. a) Uncoated module. b) Coating process. c) Carbon‐soot‐coated module. d) System set‐up and e) DC–DC boost converter.

The proposed design consists of solar radiations‐based thermoelectric energy harvesting system comprising of 1) thermoelectric module (12 706), 2) candle soot, 3) Fresnel lens of surface area 784 cm^2^, and 4) aluminium water block. The schematic of the experimental setup is shown in **Figure** **1**. The specifications of Al_2_O_3_‐based thermoelectric module (TEC‐12706) of dimensions 40 mm x 40 mm x 3.9 mm are shown in **Table** **1**. The module was coated with candle soot by directly exposing the upper flame of candle. In order to study the effect of CS coating thickness, studies were performed on five different samples. The coated samples were named as L1, L2, L3, and L4 according to increasing thickness of carbon soot while the uncoated module was named as L0. The thicknesses of candle soot coating in the coated samples L1, L2, L3, and L4 are 3, 3.5, 4.5, and 6 µm (as recorded using cross‐sectional scanning electron microscopy images (not included here). The Fresnel lens focused intense sunlight on a small area of thermoelectric module as shown in Figure 1d. A rectangular point‐focused PMMA lens with short focal length and high concentration ratio is used due to its high durability and portability. A mechanical holding assembly is placed to hold the lens at desired position for focusing sun rays. To maintain the temperature difference, the module (12 706) was placed on an aluminium water block for cooling from the bottom along with heating from the top.

**Table 1:** Specifications of thermoelectric module (12 706)

Parameter	Value
Dimensions (mm)	40 × 40 × 4.2
Weight (grams)	17
Resistance (Ω)	0.85
No. of thermocouples	127
Max. current (Amps)	6.4
Max. voltage (Volts)	14.4
Max. power (Watts)	40
Max. temperature difference, ∆*T* (°C)	68
Hot side temperature (°C)	80
Max. temperature (°C)	138

Figure 1e presents a boost converter circuit for raising the voltage level obtained from TEG to charge a 2.4 V battery. The design consists of a Schottky diode, *D* (BAT86), a 30 mH inductor (*L*), 4.7 µF capacitor (*C*_L_), and a 2N7000 MOSFET switch (*S*). The ideal lossless components will generate an output voltage *V*_out_ characterized by the duty cycle of the switching speed

(1) $${\text{V}}_{\text{out}}=\frac{{\text{V}}_{i}}{1-d}$$

where *d* is the duty cycle of the switching speed, calculated as

(2) $$d\;=\;\frac{T_{\text{on}}}{T_{\text{on}}+T_{\text{off}}}$$

Duty cycle *d* is varied using pulse width modulation (PWM) technique, where the width of the pulse, *T*_on_, is changed.

The electrical energy conversion efficiency of the thermoelectric device^[^ ^37^ ^]^ is calculated using the following equation

(3) $$\eta \;=\left(\frac{T_{\text{h}}-T_{\text{c}}}{T_{\text{h}}}\;\right)\;\left(\frac{M-1}{M+\frac{T_{\text{c}}}{T_{\text{h}}}}\right)$$

(4) $$M\;=\sqrt{1+Z\;\left(\frac{T_{\text{h}}+T_{\text{c}}}{T_{\text{h}}}\;\right)}$$

where η is the electrical conversion efficiency of the thermoelectric device, *T*_h_ is the hot‐side temperature of the thermoelectric device, *T*_c_ is the cold‐side temperature of the thermoelectric device

(5) $$Z\;=\frac{\alpha }{kR}$$

*α* is the Seeback coefficient, *k* is the thermal conductivity, *R* is the electrical resistivity

(6) $$\alpha =\frac{\text{Average}\;\text{voltage}\;\text{generated}\;\text{by}\;\text{the}\;\text{system}}{\text{Average}\;\text{temperature}\;\text{difference}\;\text{created}}$$

*k* = 1.5 W m^−1^ K^−1^ and *R* = 1.98 Ω according to data sheets of TEC‐12706.^[^ ^38^ ^]^

The MOSFET switches on/off and transfers the energy stored in the inductor to boost the voltage generated by thermoelectric module. The switch transfers the stored energy of inductor to charge the capacitor when the switch is on. Contrary, the inductor fails to perform instantaneous emf reversal in off stage resulting in transfer of accumulated energy to the capacitor. The high‐speed MOSFET controls the ON–OFF switching time and regulates the output voltage level. The open‐circuit voltage (*V*_oc_), output load current (*I*) across 50 Ω resistor, hot‐side temperature (*T*_h_), and cold‐side temperature (*T*_c_) are recorded using:

1) Electronic logging meter (Fluke 287) for recording the current and voltage.
2) Type‐K thermocouple for hot and cold temperature measurements.
3) An ordinary garden submersible pump for circulating water in water block.

**Table** **2** below gives the accuracies and ranges of the above selected instruments.

**Table 2:** Accuracy of the measuring instruments

Instrument	Parameter	Accuracy	Range
Fluke logging meter	DC current	0.05%	500 µA to 10 A
	DC voltage	0.025%	50 mA to 1000 V
Thermocouple	–	+/−2.2 °C	−40 to 260 °C

## 3 Results and Discussions

**Figure 2:**
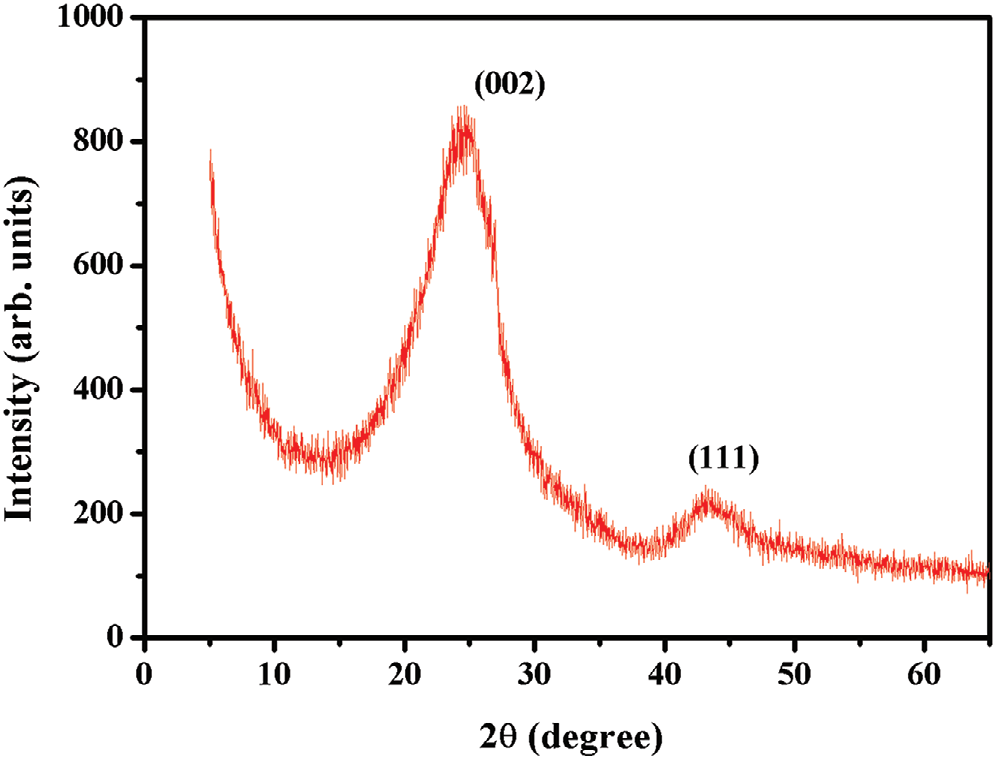
XRD pattern of CS‐coated thermoelectric module.

**Figure** **2** shows typical X‐ray diffraction (XRD) pattern (recorded using a Cu‐Kα‐based Rigaku diffractometer with 9 kW rotating anode). The high intensity peak at 24.6° confirms the presence of high amounts of amorphous carbon material. XRD is well matched with the literature.^[^ ^39^, ^40^ ^]^ The peaks at 24.6^o^ and 43.16° correspond to (002) and (111) planes, respectively, of hexagonal (graphite) structure of carbon particles.

**Figure 3:**
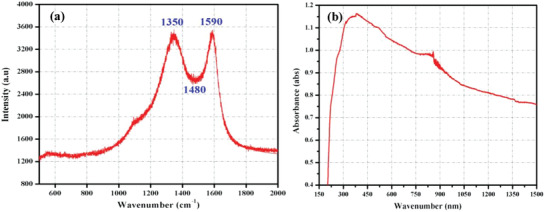
a) Raman spectrum. b) NIR‐UV‐vis spectrum of CS‐coated thermoelectric module.

**Figure** **3**a shows the Raman spectrum of incident light scattered by the irradiated molecules vibrations highlighting the crystal lattice information. The coated module was irradiated with a 535 nm green colored laser at 1800 lines per mm grating frequency from HORIBA (Model: Lab Ram Hr Evolution). At room temperature, intensity of dispersed wavelength was observed against the wave number (1/wavelength) in the range 100–2000 cm^−1^. The prominent peaks confirm with the intensity variations obtained for different carbon‐based vibration mode structures. The peak at 1350 cm^−1^ is attributed to sp^3^‐hybridized carbon, structural defects, and amorphous carbon. Another peak at 1590 cm^−1^ appears due to sp^2^‐oriented C—C stretched bonds present in soot. Weaker Raman band at 1480 belongs to certain disordered proportions of carbon material present in soot.^[^ ^40^ ^]^ Figure 3b shows the absorption of near‐IR (NIR), UV, and visible spectrum by the soot particles. A gradual increase in absorbance value is observed for spectrum range 2400–1200 nm falling in NIR region. NIR‐UV region between 1200 and 370 nm shows an appreciable increase in absorption value as compared to region below 370 nm where it decreases swiftly. Carbon particle diameter lies in the range of 30–40 nm as observed from scanning electron micrograph (not shown here).

**Figure 4:**
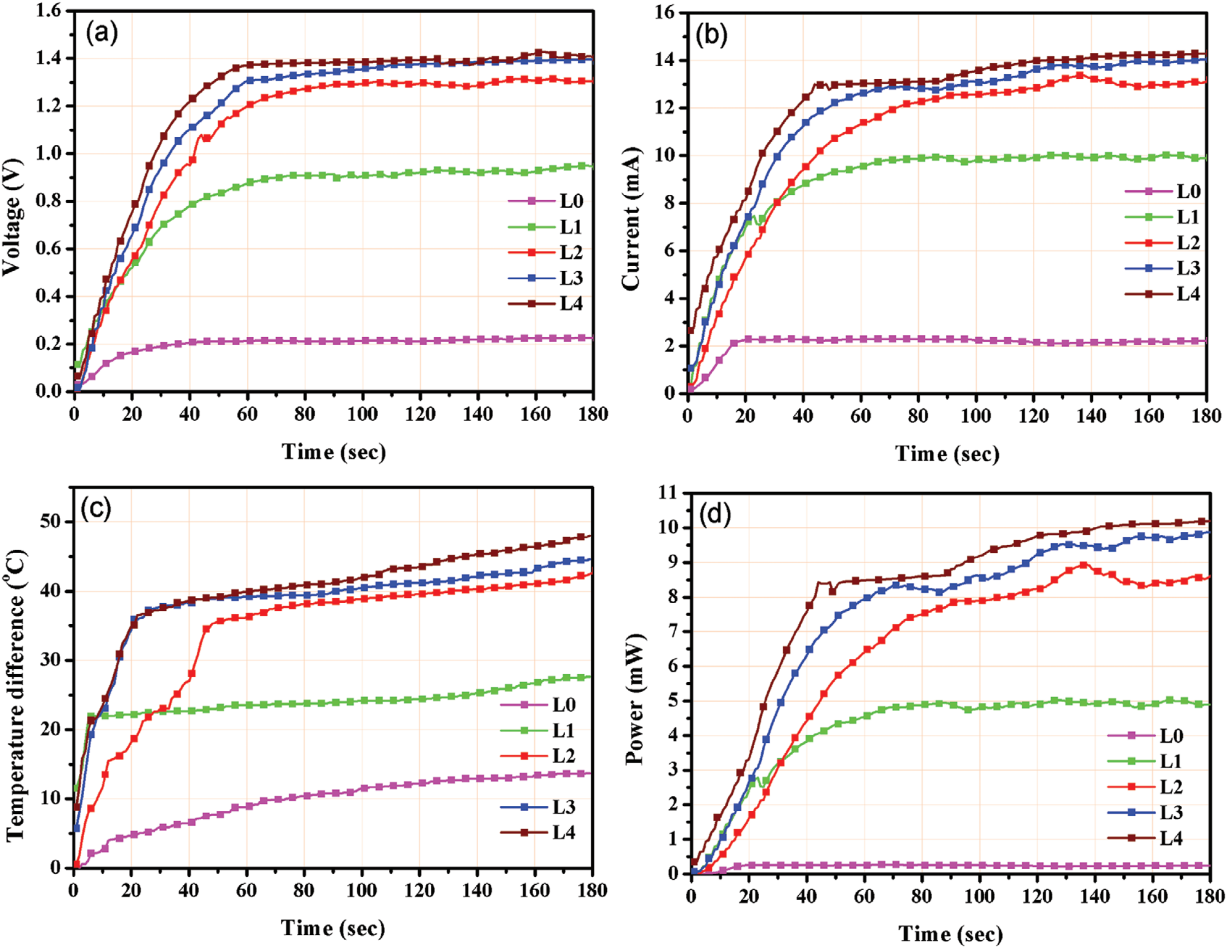
a) Open‐circuit voltage. b) Short‐circuit current. c) Temperature gradient. d) Output power.

**Figure** **4**a presents the open‐circuit voltage for uncoated and coated samples (L0 to L4 samples). It is evident that the L4 sample revealed the best results. This temporal temperature difference is largely governed by the amount of heating reaching the thermoelectric material. As the thickness of candle soot increased, the thermoelectric sample may not be able to receive thermal energy promptly which results in lower output voltage. The voltage rises impressively to 1.5 V in L4 sample while uncoated module L0 could deliver only 0.22 V. It is notable that the open‐circuit voltage across the four times coated sample rises more than six times than the uncoated sample. This increase is largely governed by the high heat absorption in the black body. Figure 4b compares the short‐circuit current across a 50 Ω load resistor for all the uncoated and coated samples understudy. Results indicate that the current increases significantly in coated thermoelectric module as compared to that of uncoated thermoelectric modules. A maximum of 14 mA of current was measured in L4 sample while only 2.2 mA current was observed across uncoated module (L0) under similar experimental conditions. An impressive increase in electrical current (more than six times) is noted due to high heat absorption across L4 sample. Figure 4c shows temperature difference across the two sides of thermoelectric module for all the uncoated and coated samples. Figure 4d shows the output power generated across a resistance of 50 Ω by the uncoated and coated modules (for 180 s). A maximum power of 10.2 mW was generated by L4 sample along with a 14 mA current output, while uncoated thermoelectric module delivered 0.24 mW of output power. Thus, a significant increase of more than 42 times is observed in four times coated sample in comparison to uncoated sample. Clearly, candle‐soot‐coated module outperformed conventional uncoated module by generating significantly higher electrical outputs.

**Figure 5:**
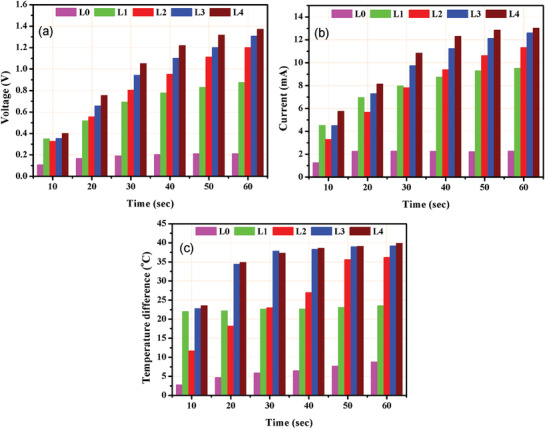
a) Open‐circuit voltage. b) Short‐circuit current. c) Temperature difference.

Comparative analysis of output voltage, current, and temperature difference values of uncoated and coated samples for 60 s is shown in **Figure** **5**. As evident from the plots, the best open‐circuit voltage is generated by L4 sample while other samples generated a lower voltage value (Figure 5a). An appreciable increase in voltage is recorded in all the coated samples as compared to uncoated sample. Also, highest short‐circuit current values shown in Figure 5b are recorded for L4 sample as compared to other cases under consideration. This increase in voltage and current is attributed to gradual increase in temperature difference values for uncoated and coated samples illustrated in Figure 5c. The corresponding values are listed in **Table** **3**. The maximum voltage reaches 1.37 V for a corresponding temperature difference of 39.9 °C, while maximum current obtained is 13.3 mA after 60 s of concentrated solar irradiation using Fresnel lens on the system.

**Table 3:** Voltage and current obtained for corresponding temperature difference during first 60 s

Time [s]	Voltage [V]					Current [mA]					Temperature difference [°C]				
	L0	L1	L2	L3	L4	L0	L1	L2	L3	L4	L0	L1	L2	L3	L4
10	0.11	0.35	0.33	0.35	0.40	1.27	4.52	3.30	4.51	5.77	2.80	22.00	11.70	22.80	23.50
20	0.17	0.52	0.56	0.66	0.75	2.27	6.97	5.69	7.30	8.14	4.70	22.20	18.20	34.40	34.90
30	0.19	0.69	0.80	0.94	1.05	2.28	7.98	7.83	9.76	10.84	5.90	22.60	23.00	37.80	37.30
40	0.21	0.78	0.95	1.10	1.22	2.27	8.76	9.41	11.24	12.32	6.50	22.70	27.00	38.30	38.60
50	0.21	0.83	1.11	1.20	1.32	2.24	9.29	10.63	12.13	12.86	7.70	23.10	35.60	39.00	39.10
60	0.21	0.88	1.20	1.31	1.37	2.28	9.53	11.32	12.61	13.03	8.80	23.50	36.20	39.20	39.90

**Figure 6:**
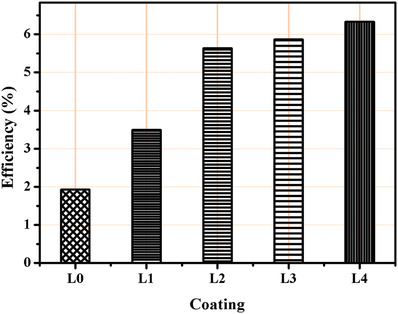
Maximum conversion efficiency (η).

To show the energy conversion efficiency of the system, **Figure** **6** shows the maximum conversion efficiency obtained for uncoated and coated samples estimated using Equation (3). The value of Seeback coefficient (α) was calculated for uncoated and coated cells individually. The efficiency of all the coated samples (L1–L4) shows significant increase as compared to uncoated sample. The average temperature difference for uncoated cell is 10 °C with maximum difference of 14 °C. An average temperature difference of 40 °C is achieved with a maximum value of 48 °C in L4 sample. A maximum conversion efficiency of 6.3% was estimated (for L4 sample) using abovementioned equation. The uncoated cell shows 1.9% efficiency for maximum 14 °C temperature difference. L1, L2, and L3 performed for maximum efficiency of 3.5%, 5.6%, and 5.9%. Clearly, coating the cell with candle soot enhanced the overall system efficiency by 3.3% making the system three times more efficient as compared to conventional uncoated cell‐based system. Further, 1.5 V obtained from L4 is boosted using a boost converter to charge a 2.4 V rechargeable VARTA battery. The output is regulated little higher than 2.4 V to charge 4.7 µF capacitor by energizing 30 mH inductor. It took 1.5 h to completely charge the battery with a constant temperature difference. System with such power levels can be considered for running ultra‐low power devices eliminating the need of continuous battery replacements. Thus, we advocate the potential of thermoelectric energy harvesting for powering low power devices and appliances thereby reducing their battery dependency.

A comparative analysis of some thermoelectric generators is presented in **Table** **4**. These results indicate that candle soot coating could be a cost‐effective technique to improve the performance of thermoelectric modules.

**Table 4:** Comparative analysis of thermoelectric generators

Ref.	Thermoelectric module	No. of modules	Coating	Concentrator	Heating source	Open‐circuit voltage [V]	Current [mA]	Power [mW]
Lin et al.^[^ ^41^ ^]^	NA	1	Cu NPs@Zn foil, Ck@membrane, RGO@Ni foam	–	Xenon lamp	0.21	1.34	0.28
Sudharshan et al.^[^ ^42^ ^]^	NA	3	Al alloy	Glass plate	Solar radiations	0.8	55.25	44.2
Ogbonnaya et al.^[^ ^43^ ^]^	1261G‐7L31‐04CQ	1	Nickel‐tin coating on Cu plate	–	Halogen lamp	0.13	70.38	9.15
Köysal et al.^[^ ^44^ ^]^	TEG1‐12611‐8.0	2	Graphene	Fresnel lens	Solar radiations	1.41	193.61	273
Sundarraj et al.^[^ ^45^ ^]^	TEG1‐127‐1.4‐1.0	6	–	Parabolic trough collector	Three 50 W electrical heater	5.7	810	4700
Estrada‐López et al.^[^ ^46^ ^]^	CP60333	1	–	Fresnel lens	Solar radiations	0.18	38.5	6.93
Present work	TEC‐12706	1	Candle soot	Fresnel lens	Solar radiations	1.5	14	10.2

## 4 Conclusions

The present work is an experimental analysis for solar energy harvesting using candle‐soot‐coated thermoelectric module. A significant temperature difference was created across two surfaces of module by concentrating sunlight and flowing water (on counter surface). Module was coated with different thickness of candle soot using direct exposure of candle flame. A maximum of 1.5 V of open‐circuit voltage and 14 mA of peak current were obtained for L4 sample. This is an appreciable increase as compared to conventional (uncoated) thermoelectric module which gives maximum voltage of 0.22 V and 2.2 mA peak current. The 10 mW harvested power is reported from soot‐coated thermoelectric module outperforming the conventional uncoated module with 0.24 mW power only. A rechargeable battery was also charged in 1.5 h to demonstrate the energy storage application. The stored energy is sufficient to run low and ultra‐low power sensors and other electronic devices. The harnessed output power can be further increased by cascading more than one such thermoelectric modules. However, the impedance matching of such arrangement should be investigated for maximum power transfer.

## Conflict of Interest

The authors declare no conflict of interest.
